# On-The-Fly Syntheziser Programming with Fuzzy Rule Learning

**DOI:** 10.3390/e22090969

**Published:** 2020-08-31

**Authors:** Iván Paz, Àngela Nebot, Francisco Mugica, Enrique Romero

**Affiliations:** Soft Computing Research Group, Intelligent Data Science and Artificial Intelligence Research Center, Computer Sciences Department, Universitat Politècnica de Catalunya—BarcelonaTech, 08012 Barcelona, Spain; ivanpaz@cs.upc.edu (I.P.); fmugica@cs.upc.edu (F.M.); eromero@cs.upc.edu (E.R.)

**Keywords:** fuzzy-rules, live coding, syntheziser programming

## Abstract

This manuscript explores fuzzy rule learning for sound synthesizer programming within the performative practice known as live coding. In this practice, sound synthesis algorithms are programmed in real time by means of source code. To facilitate this, one possibility is to automatically create variations out of a few synthesizer presets. However, the need for real-time feedback makes existent synthesizer programmers unfeasible to use. In addition, sometimes presets are created mid-performance and as such no benchmarks exist. Inductive rule learning has shown to be effective for creating real-time variations in such a scenario. However, logical IF-THEN rules do not cover the whole feature space. Here, we present an algorithm that extends IF-THEN rules to hyperrectangles, which are used as the cores of membership functions to create a map of the input space. To generalize the rules, the contradictions are solved by a maximum volume heuristics. The user controls the novelty-consistency balance with respect to the input data using the algorithm parameters. The algorithm was evaluated in live performances and by cross-validation using extrinsic-benchmarks and a dataset collected during user tests. The model’s accuracy achieves state-of-the-art results. This, together with the positive criticism received from live coders that tested our methodology, suggests that this is a promising approach.

## 1. Introduction

This manuscript explores fuzzy rule models for automatic programming of sound synthesis algorithms in the context of the performative artistic practice known as live coding [1,2].

Live coding is the act of writing source code in an improvised way to create music or visuals, arising from the computers’ processing capacities that allowed for real-time sound synthesis around the new millennium. Therefore, the phrase “live coding” implies programming sound synthesis algorithms in real time. To do this, one possibility is to have an algorithm that automatically creates variations out of a few presets A preset is a configuration of a synthesis algorithm together with a label describing the resulting sound selected by the user [3]. However, the need for real-time feedback and the small size of the data sets, which can even be collected mid-performance, act as constraints that make existent automatic synthesizer programmers and other learning algorithms unfeasible to use. Furthermore, the design of such algorithms is not oriented to create variations of a sound, but rather to find the synthesizer parameters that match a given one.

State-of-the-art automatic synthesizer programmers apply optimization algorithms that receive a target sound together with a sound synthesis algorithm and conduct a search approaching the target. For example, in [4], the “sound matching” performance of a hill climber, a genetic algorithm, and three deep neural networks (including a Long short-term memory) are compared.

At the beginning of the new millennium, diverse systems using interactive evolution were developed [5,6]. These systems represent the settings in genomes, which are then evolved by genetic algorithms that use human selection as the fitness function. Although they provide great capabilities, the selection of the sounds, as they have to be listened to, is time consuming; as such, its use in live coding is hard to manage.

Timbre is the set of properties that allow us to distinguish between two instruments playing the same note with the same amplitude. Some new approaches to timbre in sound synthesis [7] focus on models of instruments with “static” sound. Therefore, these approaches do not consider some elements of synthesizers, such as low frequency oscillators, which produce dynamic changing sounds over time (sometimes over several minutes).

In [8], a methodology is presented that relates the spaces of parameters and audio capabilities of a synthesizer in such a way that the mapping relating those spaces is invertible, which encourages high-level interactions with the synth. The system allows intuitive audio-based preset exploration. The mapping is built so that *“exploring the neighborhood of a preset encoded in the audio space yields similarly sounding patches, yet with largely different parameters.”* As the mapping is invertible, the parameters of a sound found in the audio space are available to create a new preset. The system works using a modification of variational auto-encoders (VAE) [9] to structure the information and create the mapping. By using VAE, parametric neural networks can be used to model the encoding and decoding distributions. Moreover, they do not need large datasets to be trained. This system works effectively as an exploratory tool in a similar sense to interactive-evolution based approaches. However, its interface is still oriented to sound matching and exploring rather than to automatically producing variations (it might be an interesting feature though). Furthermore, the resulting encodings are difficult to interpret from a human (especially non expert) perspective.

A deep learning based system that allows for interpolation and extrapolation between the timbre of multiple sounds is presented in [10]. Deep-learning systems are a promising path for sound synthesis applications, although their training times still do not allow for real-time feedback.

An algorithm, designed for live coding performance, that receives a set of labeled presets and creates real time variations out of them is proposed in [3]. It also allows for the addition of new input presets in real time and starts working with only two presets. The algorithm searches for regularities in the input data from which it induces a set of IF-THEN rules that generalize it. However, these rules only describe points that do not cover the whole feature space, providing little insight into how the preset labels are distributed. Here, we present an algorithm able to extend IF-THEN rules to hyperrectangles, which in turn are used as the cores of membership functions to create a map of the input feature space. For such a pursuit, the algorithm generalizes the logical rules solving the contradictions by following a maximum volume heuristic. The user controls the induction process through the parameters of the algorithm, designed to provide the affordances to control the balance between novelty and consistency in respect to the input data. The algorithm was evaluated both in live performances and by means of a classifier using cross-validation. In the latter case, as there are no datasets, we used a dataset collected during user tests and extrinsic standard benchmarks. The latter, although they do not provide musical information, do provide general validation of the algorithm.

Even though this is a purely aesthetic pursuit that seeks to create aesthetically engaging artifacts, it is surprising that the accuracy of the models reaches state-of-the-art results. This, together with the positive criticism that the performances and recordings received, suggests that rule learning is a promising approach, able to build models from few observations of complex systems. In addition, to the best of the author’s knowledge, inductive rule learning has not been explored beyond our work [3,11] neither for automatic synthesizer programming nor within live coding.

The rest of this manuscript is structured as follows: Section 2 introduces rule learning for synthesizer programming; Section 3 presents the algorithm that extends IF-THEN rules; Section 4 discusses user tests, cross-validation tests and the reception of the live performances and recordings; Finally, Section 5 contains the conclusions.

## 2. Inductive Rule Learning for Automatic Synthesizers Programming

RuLer is an inductive rule learning algorithm designed in the context of live coding for automatic synthesizers programming [3]. It takes as input a set of labeled presets, from which a set of IF-THEN rules generalizing them is obtained. Examples of labels could be: “intro” if the preset is intended to be used during the intro of a piece, or “harsh”, which could be the linguistic label describing the produced sound.The generalization process is based on the patterns found through the iterative comparison of the presets. To compare the presets, a dissimilarity function receives a pair of them and returns *True* whenever they are similar enough according to the specific form of the function and a given threshold. The dissimilarity threshold (d∈N) is established by the user. The algorithm works as follows:

- Each preset is considered an IF-THEN rule and represented as an array of size *N*. Its first N−1 entries (the rule antecedents) correspond to one parameter of the synthesis algorithm and the last entry to the label assigned to the combination (rule consequent). For example, a rule r = [{3}, {5}, intro] is read in the following way: “if the first parameter takes a value of 3 and the second a value of 5 then the preset label is intro”. A rule *r* = [{1,2,3}, {7}, . . . , {3}, intro] is read as: IF *r*[1] = 1 OR 2 OR 3 AND *r*[2] = 7 AND . . . AND *r*[N−1] = 3 THEN label = intro. The rules are stored in a list so they can be accessed by its index.
  The algorithm iterates as follows, until no new rules can be created:
  1. Take the first rule from the rule set (list).
  2. Compare the selected rule with the other rules using the dissimilarity function (Section 2.1). If a pattern is found, i.e., the rules have the same class and the dissimilarity between them is less than or equal to the threshold *d* established by the user, create a new rule using the *create_rule* function (Section 2.2).
  3. Eliminate the redundant rules from the current set. A rule $r_{1}$ is redundant with respect to a rule $r_{2}$ (of the same class) if ∀i∈ {0, . . . N−1}, $r_{1}\left[i\right]$ ⊂ $r_{2}\left[i\right]$.
- Add the created rules at the end of the rule set.

### 2.1. Dissimilarity Function

The *dissimilarity* function receives two rules ($r_{1},r_{2}$) together with a threshold d∈N and returns *True* if the rules have the same category and *dissimilarity*$(r_{1},r_{2})\leq d$. It returns *False* otherwise. The parameter *d* is an input parameter of the algorithm.

The *dissimilarity* function, currently implemented in the RuLer algorithm, counts the number of empty intersections between the sets of the corresponding entries in the rules. For example, if $r_{1}$ = [{1}, {3,5}, intro] and $r_{2}$ = [{1,3}, {7,11}, intro], *dissimilarity*($r_{1}$,$r_{2}$) = 1. If $r_{1}$ = [{1}, {3,5,7}, intro] and $r_{2}$ = [{1,3}, {7,11}, intro], *dissimilarity*($r_{1}$,$r_{2}$) = 0.

### 2.2. Create_Rule Function

This functions receives pairs of rules $r_{1},r_{2}$, satisfying that *dissimilarity*$(r_{1},r_{2})\leq d$. Then, it creates a new rule according to the way it is defined. The function currently used creates a new rule by taking the unions of the corresponding sets of the rules received. For example, if $r_{1}$ = [{1}, {3,5,7}, intro] and $r_{2}$ = [{1,3}, {7,11}, intro], then $r_{1,2}$ = [{1,3}, {3,5,7,11}, intro]. The candidate rule is accepted if the following conditions are met:

1. No contradictions (i.e., rules with the same parameter values but different label) are created during the generalization process.
2. From all the presets contained in the candidate rule, the percentage of them contained in the original data are greater than or equal to a ratio∈ [0,1]. This number is also an input parameter of the algorithm defined by the user. For instance, ratio = 1 implies that 100% of the instances contained in a candidate rule have to be present in the input data for the rule to be accepted. ratio=0.5 needs 50% of the instances, etc.

### 2.3. Domain Specific Functions

Note that the *dissimilarity* and *create_rule* functions can be changed according to the objects being compared and the desired generalization. For example, for harmonic objects, we probably want to use a dissimilarity that looks at the harmonic content. For rhythms, temporal factors need to be addressed. See, for example, [12], for a comparison of rhythmic similarity measures.

### 2.4. RuLer Characteristics

The RuLer algorithm is designed to return all the existing patterns, expressing as rules all pairs of instances satisfying $dissimilarity(r_{1},r_{2})\leq d$, as its main intention is to offer all possibilities for creating new instances. Therefore, it is possible for a single instance, let us call it $r_{2}$, to be included in more than one valid rule if $r_{1},r_{2}$, and $r_{3}$ are single rules satisfying that: $dissimilarity(r_{1},r_{2})\leq d$, $dissimilarity(r_{2},r_{3})\leq d$ and $dissimilarity(r_{1},r_{3})$ >d.

To illustrate this case, consider the dataset of Table 1.

**Figure 1:**
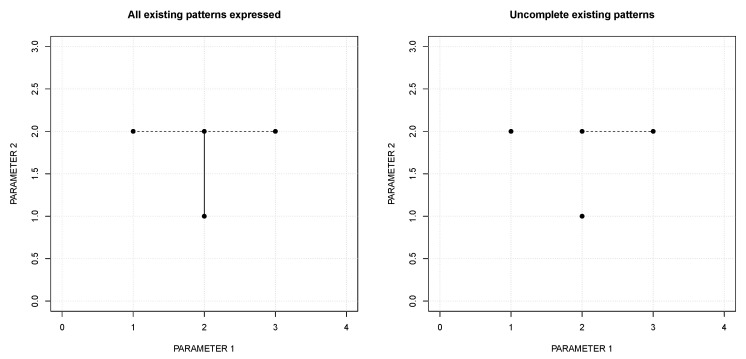
Resulting rules using data of Table 1 with their possible extensions in a solid line and a dashed line. Left, extracted by the RuLer algorithm with parameters d=1 and ratio=1. Right, extracted by using the Hamming distance d=1 and, whenever a pattern is found, creating a new rule by taking the unions of the parameter values and eliminating the component rules.

The RuLer algorithm with parameters d=1 and ratio=1 produces the rules: [{2}, {1, 2}, ‘intro’], [{1, 2, 3}, {2}, ‘intro’]. These rules are shown, with their possible extensions in a solid line and a dashed line respectively, at the left of Figure 1.

Notice that the combination [{2},{2},‘intro’] is present in both rules. As mentioned, if this were not the case, one of the patterns might fail to return to the user. To illustrate this, consider the same dataset and let us use the Hamming distance (d=1) as the similarity function. Then, suppose that the create_rule function, whenever a pattern is found, creates a rule taking the unions of the parameters of the respective rules and eliminates the component rules after producing the new one. With these conditions, comparing $r_{1}$ and $r_{2}$ produces the rule $r_{1,2}$ [{2,3},{2},‘intro’]. This rule will not produce another rule when compared with the remaining data: $r_{3}$ [{1},{2},‘intro’] and $r_{4}$ [{2},{1},‘intro’]. Therefore, the resulting rule set is: $r_{1,2}$ [{2,3}{2},‘intro’], $r_{3}$ [{1},{2},‘intro’] and $r_{4}$ [{2},{1},‘intro’]. This is shown at the right of Figure 1. The resulting rule set does not express the existing patterns between [{2},{1},‘intro’] and [{2},{2},‘intro’] as well as between [{1},{2},‘intro’] and [{2},{2},‘intro’] or [{3},{2},‘intro’]. To avoid this, the create_rule and the dissimilarity function were conceived to return all the patterns found in the data.

Regarding how *d* and ratio work, consider the simple set of individual rules presented in Table 2.

If d=2 and ratio=1/4, the single rule that models the dataset is at the mid part of Table 2. The number of allowed empty intersections among the single rules at the Top of the Table is two. Then, every pair of rules can be compacted into a new rule during the process. As the ratio of single rules that have to be contained in the original data for any created rule is 1/4, the rule at the mid part can be created as it contains all the instances in the original data which are 1/3 of the number of single instances of the rule (nine). Note that this is true if all seen values are: for the first attribute 1, 2, and 3; For the second attribute 4, 5, and 6; For the third attribute 6.

If d=2 and ratio=1/2, the rule model extracted by the algorithm is presented at the bottom of Table 2. Here, the ratio of single instances contained in any rule that have to be in the original data are 1/2. Therefore, the rule at the middle of Table 2 cannot be created.

The parameter ratio is constant because it defines the level of generalization that the user of the algorithm wants to explore. The ratio allows for the extension of the knowledge base to cases that have not been previously used to build the model. If the user is more conservative, the ratio should be closer to 1. If the goal is to be more exploratory, lower ratios are needed.

Finally, although no comparisons of computational time were carried out, the algorithm complexity serves to estimate its performance. If *m* is the size of input data, the algorithm complexity is O(m∗(m−1)). This complexity considers the dissimilarity and create_rule functions described. This complexity is better than a previous version of the algorithm $O(2^{m}-1)$ presented in [11].

## 3. FuzzyRuLer Algorithm

The FuzzyRuLer algorithm constructs a fuzzy rule set of trapezoidal membership functions out of logical IF-THEN rules. For that, it builds hyperrectangles (Section 3.1), which are the cores of the trapezoidal membership functions and, in turn, are used to fit the supports (Section 3.2).

### 3.1. Building Cores

**Figure 2:**
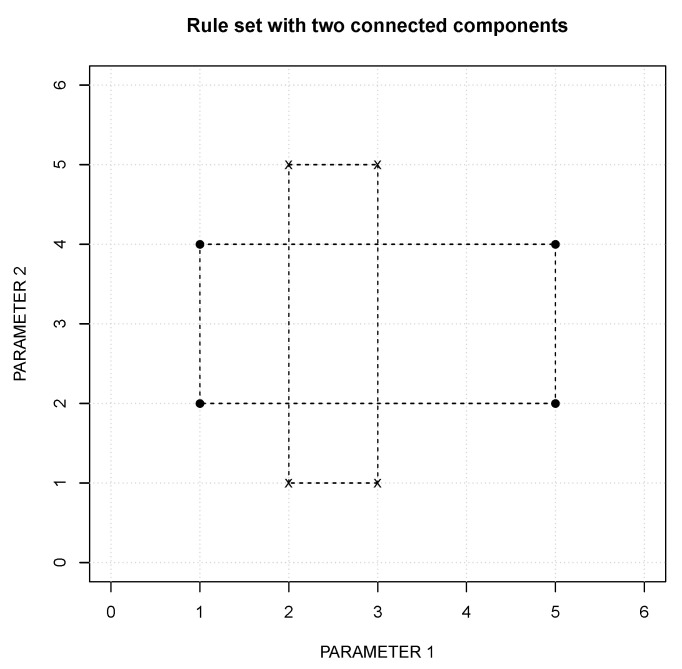
Rule [[2,3], [1,5], harsh] intersects rule [[1,5], [2,4], calm]. Harsh is represented by an “x” and Calm by a “.” in the plot.

**Figure 3:**
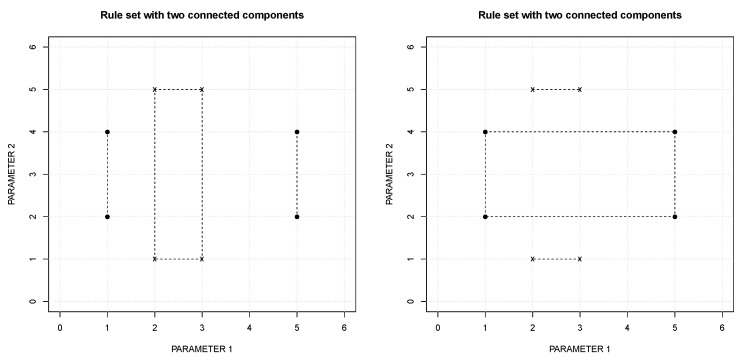
Two possible ways of resolving the contradiction that appears in Figure 2.

To build the cores, the algorithm extends the **sets** contained at the entries of the logical IF-THEN rules to **intervals** between their respective minimum and maximum values. For example, $r_{1}$ = [{1,4}, {3,5}, intro] is extended to $r_{1}$ = [[1,4], [3,5], intro], including all the values in between 1 and 4 as well as between 3 and 5. Then, instead of four values, we have a region to choose from! Next, the contradictions that might appear between the created intervals are resolved. A contradiction appears when two rules with different labels or classes intersect each other. Two rules $r_{1}$ and $r_{2}$ intersect if for all *i* (i.e., parameter placed at position *i* in the antecedent of the rule) there exists *x* in $r_{1}\left[i\right]$ such that $y_{1}\leq x\leq y_{2}$ with $y_{1}$, $y_{2}$ $\in r_{2}\left[i\right]$. If two rules with different classes intersect, it is enough to “break” one parameter to resolve the contradiction. For example, the contradiction between the rules $r_{1}$ and $r_{2}$ (at the top of Table 3 and depicted in Figure 2) can be resolved either as shown on the left or on the right of Figure 3.

To select the partition, the Measure of each set of rules is calculated and the one with maximum value is selected. The set with maximum Measure value is selected as it is the one that covers a wider region of the feature space. While the inductive process of the RuLer algorithm is intended to create new points, the generalization process of the FuzzyRuLer covers the entire observed space. Therefore, maximum coverage is the goal. The Measure of a single rule has components: *Extension* (*E*) and *dimension*, defined in Equation (1):

(1) $$\begin{array}{c} E=\sum_{i=0}^{N-1}E_{i},\mathrm{where}E_{i}=\left|max_{i}-min_{i}\right|\\ dimension=\mathrm{Number}\;\mathrm{of}\;E_{i}\;\mathrm{such}\;\mathrm{that}\;E_{i}\neq 0. \end{array}$$

In Equation (1), for each parameter *i* in the rules, $E_{i}$ is the absolute value between its maximum and minimum values. For example, if r[i] = {11,13,15}, then $E_{i}$ = 4, which is |15−11|. If r[i] = {3}, then $E_{i}$ = 0.

The *Measure* of a set of rules collects the individual measures of the rules, adding those who have the same dimension. It is expressed as an array containing the extension for each dimension. When two measures are compared, the greatest dimension wins. For example, (*Extension* = 1, *dimension* = 2) > (*Extension* = 4, *dimension* = 1). In the same way (*Extension* = 1, *dimension* = 3) > (*Extension* = 100, *dimension* = 2; *Extension* = 100, *dimension* = 1). Table 4 presents an example.

### 3.2. Fuzzy Rule Supports

**Figure 4:**
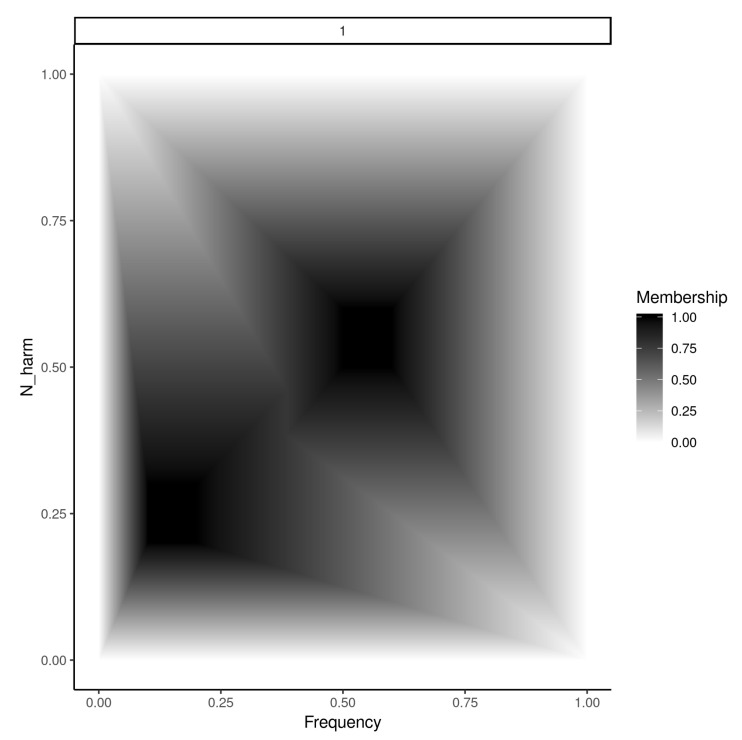
Two fuzzy rules (scaled into [0,1]) of a hypothetical Category 1 (shown at the top of the graph). The *x*-axis represents the frequency of an oscillator and the *y*-axis the number of upper harmonics added to it. The membership of a point **(Frequency, N_harm)** to Category 1 is indicated by the Membership scale at the right of the graph.

Once the cores are known, there are many possibilities for building the supports of trapezoidal membership functions. Here, as the algorithm is designed for real performance, we construct the supports using the minimum and maximum values observed for each variable. In this way, the slopes of each trapezoidal membership function are defined automatically by how close the core is to the respective minimums and maximums. Thus, each rule covers the whole observed space and the supports are defined automatically by the cores avoiding costly procedures that iteratively adjust the supports while the information is processed. This is done in the following way: For each parameter, the minimum and maximum values observed are calculated. If the parameter values are normalized, these values are 0 and 1. Then, the algorithm connects the extremes of each core with the respective minimum and maximum values of each parameter. See Figure 4 for an example.

## 4. Evaluation

Evaluation of automatic synthesizer programmers has followed two main approaches: user tests, in which expert musicians are interviewed after using the algorithm; In addition, similarity measures in sound matching tasks, in candidate sound, is compared with the target.

Let us consider the unsupervised software synthesis programmer “SynthBot” [13], which uses a genetic algorithm to search for a target sound. The search is guided by measuring the similarity of the current candidate and the target, using the sum of squared errors between their MFCCs. The system was evaluated “technically to establish its ability to effectively search the space of possible parameter settings”. Then, musicians competed with SynthBot to see who was the most competent sound synthesizer programmer. The sounds proposed by SynthBot and the musicians were compared with the target by using sound similarity measures.

In [4], a hill climber, a genetic algorithm, and three deep neural networks are used for sound matching. The results are evaluated by calculating the error score associated with the euclidean distance between the MFCCs of the proposed sound and the MFCCs of the target.

In our case, the evaluation includes: 1. The analysis of how the model generalizes a user test dataset. This evaluation is reinforced by other extrinsic benchmarks (Section 4.2). 2. The evaluation of the performances where the project has been presented and the lists where the compositions made with the algorithms have been included (Section 4.4). As one of the objectives of the FuzzyRuLer algorithm is to provide new presets classified with the same labels of the input data, the generalization using the user-labeled data are evaluated by cross-validation. The classifier used for that purpose is presented next. When the rules are used to classify new instances, the classifier assigns to them the label that it will assign to the same combinations if the model is used to produce new presets (data). In addition, cross-validation allows for the assessment of the performance of the algorithm using benchmarks in a task for which datasets might not exist.

### 4.1. Fuzzy Classifier

To classify a new preset $P=(v_{1}$, . . . , $v_{N-1})$, proceed as follows: For each rule $r_{k}$, calculate the membership of each feature value i.e., $\mu_{k,i}\left(v_{i}\right)$. Then, calculate its firing strength $\tau_{k}\left(P\right)$, which measures the degree to which the rule matches the input parameters. It is defined as the minimum of all the membership values obtained for the parameters (see Equation (2)), i.e,

(2) $$\tau_{k}\left(P\right)=min\;\left\{\;\mu_{k,i}\left(v_{i}\right)\;\right\}$$

Once the firing strength has been calculated for all rules, the assigned class will be equal to the class of the rule with maximum firing strength, as in Equation (3):

(3) $$Class\left(P\right)=\mathrm{Class}\;\mathrm{of}\;R_{c}\;\mathrm{where}\;C=\underset{k}{arg\;max}\;\left\{\tau_{k}\left(P\right)\right\}$$

**Figure 5:**
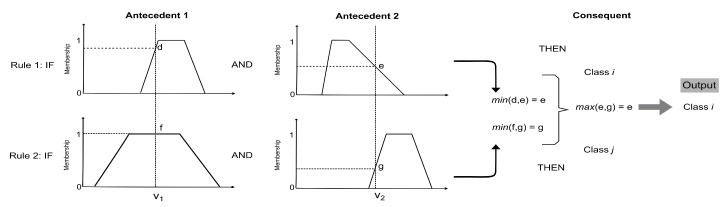
Example of classification process for a system with two rules and two parameters. The new combination $P=(v_{1},v_{2})$. For the first rule $\mu (v_{1})=d$ and $\mu (v_{2})=e$. The minimum of these values is *e*. For the second rule $\mu (v_{1})=f$, $\mu (v_{2})=g$ and min(f,g)=g. Finally, max(e,g)=e and therefore the class assigned to the instance is Classi.

An example of the classification process for a hypothetical system with two rules each with two parameters is shown in Figure 5.

### 4.2. Cross-Validation

**Figure 6:**
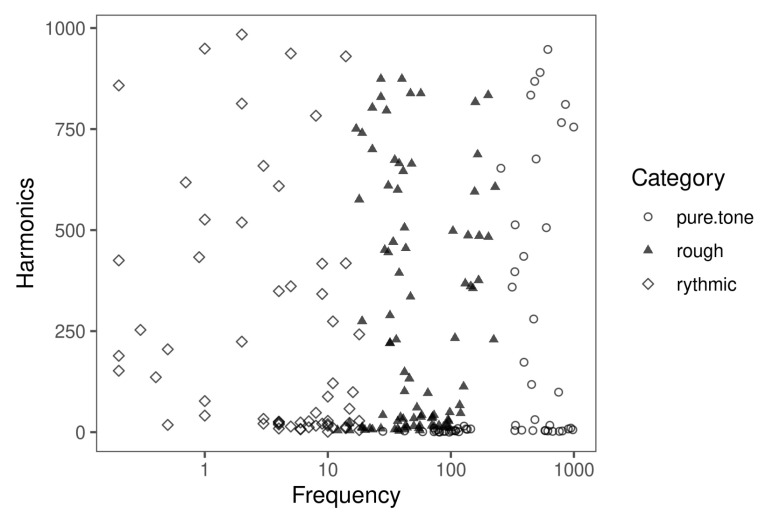
Band Limited Impulse Oscillator (Blip) data set. The *x*-axis shows the log of the fundamental frequency of the impulse generator. The *y*-axis shows the number of upper harmonics that are added to the fundamental frequency. The categories associated with the combinations (*rhythmic*, *rough* or *tone*) are shown at the right side of the graph.

To test how the algorithm models the feature space of a synthesis algorithm, we used the data set described in [11]. This dataset was generated by user tests, in which different configurations of a Band Limited Impulse Oscillator [14] were programmed by users and tagged either as *rhythmic*, *rough* or *pure tone*. For this, the users tweaked the device parameters of the synthesis algorithm: Fundamental Frequency and Number of Upper Harmonics (which are add to the fundamental frequency). Then, the parameter combinations that produced any of the searched categories were saved together with the corresponding label. The data set is shown in Figure 6.

In addition, four datasets from the UCI repository [15] were selected. As they belong to diverse domains and have different unbalanced degrees, they provide a general idea of how the algorithm behaves.

The results of the fuzzy classifier of Section 4.1 were compared with K-Nearest Neighbours, Support Vector Machine (with kernels linear, polynomial degree 2 and rbf), and Random forest classifiers.

The K-Nearest Neighbours does not require a training period (these types of algorithms are known as instance based learners). It stores the training data and learns from it (analyzes the data) as it performs real-time predictions. While this has some disadvantages (for example it is sensitive to outliers), it also makes the algorithm much faster than those that require training, such as SVM. By assigning the classes only by looking at the neighbors, new data can be added with little impact to its accuracy. These characteristics make KNN very easy to implement and to interpret (only two parameters are required: the value of K and the distance function).

The Support Vector Machine (SVM) is an algorithm with good generalization capabilities and nonlinear data handling using the kernel trick. In addition, small changes in the data do not affect its hyperplane. However, choosing an appropriate Kernel function is difficult and the algorithmic complexity and memory requirements are very high. As a consequence, it has long training times. In addition, the resulting model is difficult to interpret.

The Random Forest is based on the bagging algorithm and uses an Ensemble Learning technique. It creates many trees and combines their outputs. In this way, it reduces the overfitting problem of decision trees and reduces the variance, improving the accuracy. It handles nonlinear parameters efficiently. However, as it creates lots of trees, it requires computational power and resources. Using the Random Forest to compare is interesting because these algorithms are normally considered the alternative to rule learning. However, while a random forest algorithm might indeed perform as easy and fast as the FuzzRuler, its only parameter, **the Number of trees**, is not as expressive and interpretable for the user as parameters *d* and ratio for controlling the induction process.

Together, these algorithms provide a spectrum to compare the classifier. For each dataset, the model parameters producing the highest 10-fold (70% training and 30% test) cross-validation accuracy were selected. For the SVM, tested parameter values for C and gamma were respectively [0.01, 0.1, 1, 10, 100, 1000] and [1, 0.1, 0.01, 0.001, 0.00001, 0.000001, 10]. For KNN, the tested N values were [1, 2, 3, 4, 5, 6, 7, 8, 9, 10] and for the Random forest [1, 10, 100, 500,1000] trees were considered. In the case of the FuzzyRuLer, *d* was explored from 1 to half the number of features in the dataset and ratio with [0.9, 0.8, 0.7, 0.6, 0.5] values. Table 5 presents for each model the parameter selected and the accuracy obtained.

#### Cross Validation Results

Table 5 shows the cross-validation mean accuracy results obtained for each classifier and dataset. Table 6 presents the general mean and standard deviation for each classifier. These results show that the FuzzyRuLer yields similar results to those achieved by state-of-the-art classification algorithms. There exists abundant literature applying different machine learning algorithms to the UCI datasets; see, for instance, [16]. However, the algorithms are used for a variety of purposes and under different conditions. For example, their evaluations use different partition schemes or sometimes are performed using techniques that trade execution time to gain accuracy, e.g., leave-one-out. Here, some references intended to frame the obtained results are presented. However, the reader has to keep in mind that these experiments are not completely comparable.

For the **Wine** dataset, according to [15], the classes are separable, though only RDA has achieved 100% correct classification. The reported results are RDA: 10 0%, QDA 99.4%, LDA 98.9%, 1NN 96.1% (z-transformed data), in all cases, the results have been obtained using the leave-one-out technique.

In [17], using the **Wine-quality-red** dataset with a tolerance of 0.5 between the predicted and the actual class, the SVM best accuracies for this dataset were around 57.7% to 67.5%.

For the **Glass** dataset, [16] report the following accuracy results: KNN 0.6744, SVM 0.7442, and Large Margin Nearest Neighbors (LMNN) 0.9956.

Finally, for the **Ionosphere** dataset, in [18], Deep Extreme Learning Machines (DELM) were used for classification. According to the report, the multilayer extreme learning machine reaches an average test accuracy of 0.9447±0.0216, while the DELM reaches an average test accuracy of 0.9474±0.0292. In [16], they report the following results KNN 0.8, SVM 0.8286, LMNN 0.9971.

To compare if mean accuracies are significantly different between algorithms, we performed a statistical test. As the predictor variables are categorical and their outcomes are quantitative, we performed a comparison of means test. As there are more than two groups being compared, but there is only one outcome variable, the statistical test is the one-way-ANOVA.

Table 7 shows that the *p*-value of the one-way analysis of variance is greater than the significance level 0.05, from which we conclude that there are not significant differences between the groups. The Tukey multiple comparisons of means yields 95% family-wise confidence level. Together, these results suggest that the fuzzy model could be used to generate new instances.

### 4.3. Extracted Rules

**Figure 7:**
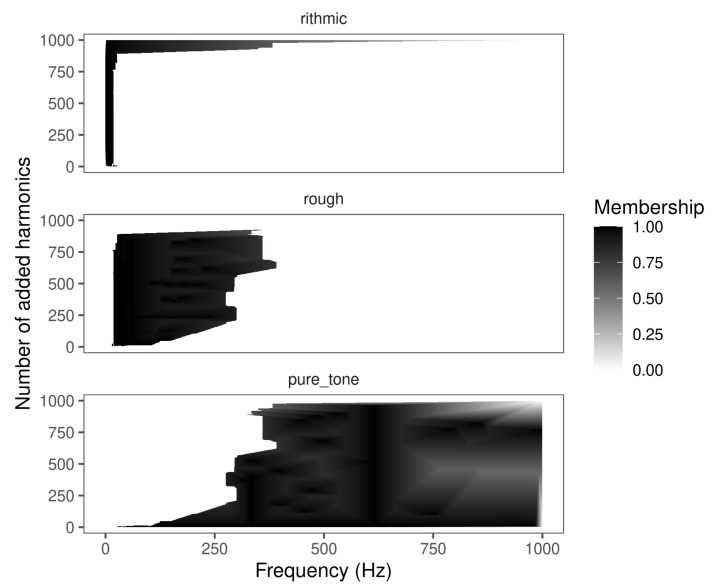
Extracted fuzzy rules for the three categories of the blip data set. The degree of membership to the class is shown at the right side of the image.

Figure 7 shows the fuzzy rules obtained for the three categories of the “Blip’’ data set (shown in Figure 6) by using the FuzzyRuLer algorithm.

Although the Blip is a simple data set, it provides insight into the algorithm capacities for identifying the underlying structures that codify the categories. In Figure 7, it can be seen that the ranges in the frequency that separate the categories are consistent with the perception thresholds described in [19]. These are: from 0 Hz to approximately 20 Hz the category is *rhythmic* no matter the number of harmonics added. From 20 Hz depending on the number of harmonics added, the sensation is *rough* until approximately 250 Hz. If the frequency is greater than 20 Hz and there are no harmonics added, or if the frequency is greater than approximately 250 Hz, the sensation is *pure tone*.

### 4.4. Live Performances and Recordings

A series of live coding performances and recordings have accompanied the design and testing of the algorithm. These have been developed in different contexts and venues including universities, artistic research centers, theatres, online streaming, smoky bars, etc.

They allow for the evaluation of: 1. The algorithm affordances and capacities to produce “interesting variations” over the input data during the performance. 2. How the community receives the music generated using the algorithms.

The live performance presented during the **live coding => music;** seminar [20], held at the Instituto Nacional de Matemática Pura e Aplicada (National Institute for Pure and Applied Mathematics) of Rio de Janeiro, is presented in [21]. The online performance presented during the **EulerRoom Equinox 2020**, which featured 72 h of live coding performances around the world (20–22 March), can be foud in [22].

The EP studio album **Visions of Space** [23], featured by the Berliner record label *Bohemian drips*, applied IF-THEN rules to generate the sections of tracks 4 and 5.

Although a subjective appreciation, the algorithm has shown effective capacities to produce new interesting material on-the-fly. The current version allows for the preloading of data before the performance and/or the saving of new instances as they are found. If all the instances are captured in real time, the space exploration process becomes part of the performance. The current implementation does not overwrite the input data with the extracted model, so the performer can extract different sets using different combinations of *d* and ratio while conducting the piece.

In 2018, the **Bandcamp Daily** featured the album Visions of the Space together with nine other albums realized during 2017 under the list *Meet the Artists Using Coding, AI, and Machine Language to Make Music* [24].

## 5. Conclusions

Real-time synthesizer programming in live coding imposes challenges to the intended use of learning algorithms, which provide numerous well-chosen examples, and have processes for data cleaning, learning and testing before selecting the final model.

Here, on the contrary, the examples are collected in real time, sometimes including musician mistakes that have to be managed as *glitches* and integrated into the performance. In cases when the data are pre-selected, the size of the datasets may be small. In other words, in this artistic practice, although it is also possible to include already trained models, the artists focus on having real-time feedback, creating the dataset mid-performance. Then, real-time algorithms that operate with small noisy data are also needed.

Inductive rule learning has offered interesting results within this context. However, the number of inducted instances is reduced and the resulting IF-THEN rules provide a poor visualization of the space. The fuzzy rule learning algorithm presented in this manuscript is able to build fuzzy rule models of the feature space out of a set of IF-THEN rules. The resulting set provides an image of the class distribution in the feature space that helps musicians to have a quick insight into the inner workings of the synthesis algorithm. As the new examples only modify the rules that they “touch”, the general model can manage outliers, integrating them into the model. The model has been evaluated during live performances and recordings which have been well-received by the community. The performances and reviews are available as part of the references. Finally, the model was also evaluated using cross-validation, comparing its results with those obtained by KNN, SVM (linear, polynomial degree 2 and rbf), and Random Forest classifiers. The one-way analysis of variance shows that there exist no significant differences among the algorithms. These results together suggest that the algorithm is a promising approach to be used in contexts, such as live coding, where the focus is not necessarily placed in model accuracy but, for example, in having real-time feedback of the algorithmic process.

## Figures and Tables

**Table 1:** Dataset to illustrate instances that appear in more than one rule.

Rule	Parameter 1	Parameter 2	Class
$r_{1}$	{3}	{2}	intro
$r_{2}$	{2}	{2}	intro
$r_{3}$	{1}	{2}	intro
$r_{4}$	{2}	{1}	intro

**Table 2:** Dataset to illustrate instances that appear in more than one rule.

Data Set			
{1}	{4}	{6}	‘a’
{2}	{5}	{6}	‘a’
{3}	{6}	{6}	‘a’
Rule set extracted with d=2 ratio=1/4			
{1, 2, 3}	{4, 5, 6}	{6}	‘a’
Rule set extracted with d=2 ratio=1/2			
{1, 2}	{4, 5}	{6}	‘a’
{1, 3}	{4, 6}	{6}	‘a’
{2, 3}	{5, 6}	{6}	‘a’

**Table 3:** The contradiction between $r_{1}$ and $r_{2}$ can be resolved by “breaking” one parameter.

Rule	Parameter 1	Parameter 2	Class
$r_{1}$	[1,5]	[2,4]	calm
$r_{2}$	[2,3]	[1,5]	harsh
**First Partition**			
$r_{1a}$	[1]	[2,4]	calm
$r_{2}$	[2,3]	[1,5]	harsh
$r_{1b}$	[5]	[2,4]	calm
**Second Partition**			
$r_{1}$	[1,5]	[2,4]	calm
$r_{2a}$	[2,3]	[1]	harsh
$r_{2b}$	[2,3]	[5]	calm

**Table 4:** Example of *extension (E)* and *dimension (dim)* for a set of rules. Note that rules with different categories contribute to the global Measure.

Rules and Measures	Parameter Values and Category		
	*Parameter 1*	*Parameter 2*	*Category*
rule $r_{1}a$	[1]	[2,4]	calm
Measure $r_{1}a$	$E_{1}$ = 0	$E_{2}$ = 2	*E* = 2, *dim* = 1
rule $r_{2}$	[2,3]	[1,5]	harsh
Measure $r_{2}$	$E_{1}$ = 1	$E_{2}$ = 4	*E* = 5, *dim* = 2
rule $r_{1}b$	[5]	[2,4]	calm
Measure $r_{1}b$	$E_{1}$ = 0	$E_{2}$ = 2	*E* = 2, *dim* = 1
**Measure: *E* = 5, *dim* = 2; *E* = 4, *dim* = 1**			
rule $r_{1}$	[1,5]	[2,4]	calm
Measure $r_{1}$	$E_{1}$ = 4	$E_{2}$ = 2	*E* = 6, *dim* = 2
rule $r_{2}a$	[2,3]	[1]	harsh
Measure $r_{2}a$	$E_{1}$ = 1	$E_{2}$ = 0	*E* = 1, *dim* = 1
rule $r_{2}b$	[2,3]	[5]	harsh
Measure $r_{2}b$	$E_{1}$ = 1	$E_{2}$ = 0	*E* = 1, *dim* = 1
**Measure: *E* = 6, *dim* = 2; *E* = 2, *dim* = 1**			

**Table 5:** Data sets Wine, Wine-quality-red, Glass and Ionosphere, selected from the UCI repository [15]. The Blip data set was obtained from [11]. The accuracy was calculated using 10-fold cross validation.

Data	Algorithm	Parameters	Mean Accuracy 10-fcv
Wine	SVM linear kernel	best C = 0.1	0.9717
	KNN	neighbors = 1	0.7514
	RANDOM FOREST	trees = 100	0.9830
	FuzzyRuLer	d = 9; ratio = 0.7	0.9554
	SVM poly 2	C = 0.01; gamma = 1	0.9717
	SVM rbf	C = 1000; gamma = 1 × 10${}^{-5}$	0.9378
Wine-quality-red	SVM linear kernel	C = 100	0.6
	KNN	neighbors = 9	0.5475
	RANDOM FOREST	trees = 10	0.59
	FuzzyRuLer	d = 1; ratio = 0.5	0.6204
	SVM poly 2	C = 0.01; gamma = 0.001	0.64
	SVM rbf	C = 1; gamma = 0.1	0.66
Glass	SVM linear kernel	C = 1000	0.6384
	KNN	neighbors = 6	0.6760
	RANDOM FOREST	trees = 1000	0.6572
	FuzzyRuLer	d = 6; ratio = 0.8	0.6636
	SVM poly 2	C = 0.1; gamma = 1	0.6666
	SVM rbf	C = 10; gamma = 0.1	0.6854
Ionosphere	SVM linear kernel	C = 10	0.8857
	KNN	neighbors = 1	0.86
	RANDOM FOREST	trees = 1000	0.9342
	FuzzyRuLer	d = 6; ratio = 0.5	0.9033
	SVM poly 2	C = 0.1; gamma = 1	0.92
	SVM rbf	C = 10; gamma = 0.1	0.9485
Blip	SVM linear kernel	C = 1	0.8097
	KNN	neighbors = 4	0.8195
	RANDOM FOREST	trees = 500	0.8585
	FuzzyRuLer	d = 2; ratio = 0.8	0.8690
	SVM poly 2	C = 0.1; gamma = 0.1	0.89
	SVM rbf	C = 1; gamma = 0.1	0.775

**Table 6:** Mean and standard deviation achieved for each classifier considering all the datasets.

Classifier	Mean	sd
FuzzyRuLer	0.802	0.150
KNN	0.731	0.124
Random-forest	0.805	0.173
SVM-linear-kernel	0.781	0.159
SVM-poly-2	0.818	0.153
SVM-rbf	0.801	0.136

**Table 7:** One-way analysis of variance of the means shown in Table 6.

	Df	Sum Mean	Sq	Fvalue	Pr (>F)
Classifier	5	0.0242	0.004832	0.214	0.953
Residuals	24	0.5408	0.022532		
